# Development of NK cell-based cancer immunotherapies through receptor engineering

**DOI:** 10.1038/s41423-024-01145-x

**Published:** 2024-03-05

**Authors:** Audrey Page, Nicolas Chuvin, Jenny Valladeau-Guilemond, Stéphane Depil

**Affiliations:** 1 Centre de Recherche en Cancérologie de Lyon, UMR INSERM U1052 CNRS 5286, Centre Léon Bérard, Lyon, France; 2 ErVimmune, Lyon, France; 3 Centre Léon Bérard, Lyon, France; 4 Université Claude Bernard Lyon 1, Lyon, France

**Keywords:** NK cells, Cell therapy, Receptor engineering, CAR, Oncology, Immunology

## Abstract

Natural killer (NK) cell-based immunotherapies are attracting increasing interest in the field of cancer treatment. Early clinical trials have shown promising outcomes, alongside satisfactory product efficacy and safety. Recent developments have greatly increased the therapeutic potential of NK cells by endowing them with enhanced recognition and cytotoxic capacities. This review focuses on surface receptor engineering in NK cell therapy and discusses its impact, challenges, and future directions.

Most approaches are based on engineering with chimeric antigen receptors to allow NK cells to target specific tumor antigens independent of human leukocyte antigen restriction. This approach has increased the precision and potency of NK-mediated recognition and elimination of cancer cells. In addition, engineering NK cells with T-cell receptors also mediates the recognition of intracellular epitopes, which broadens the range of target peptides. Indirect tumor peptide recognition by NK cells has also been improved by optimizing immunoglobulin constant fragment receptor expression and signaling. Indeed, engineered NK cells have an improved ability to recognize and destroy target cells coated with specific antibodies, thereby increasing their antibody-dependent cellular cytotoxicity. The ability of NK cell receptor engineering to promote the expansion, persistence, and infiltration of transferred cells in the tumor microenvironment has also been explored. Receptor-based strategies for sustained NK cell functionality within the tumor environment have also been discussed, and these strategies providing perspectives to counteract tumor-induced immunosuppression.

Overall, receptor engineering has led to significant advances in NK cell-based cancer immunotherapies. As technical challenges are addressed, these innovative treatments will likely reshape cancer immunotherapy.

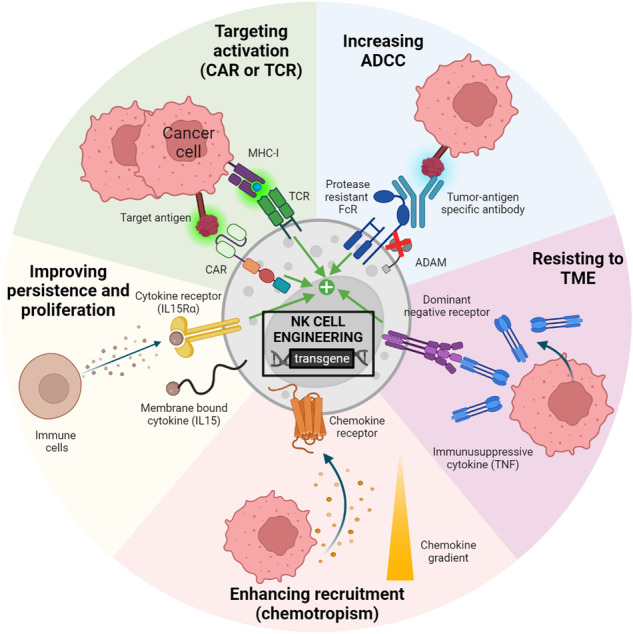

## Introduction

**Fig. 1:**
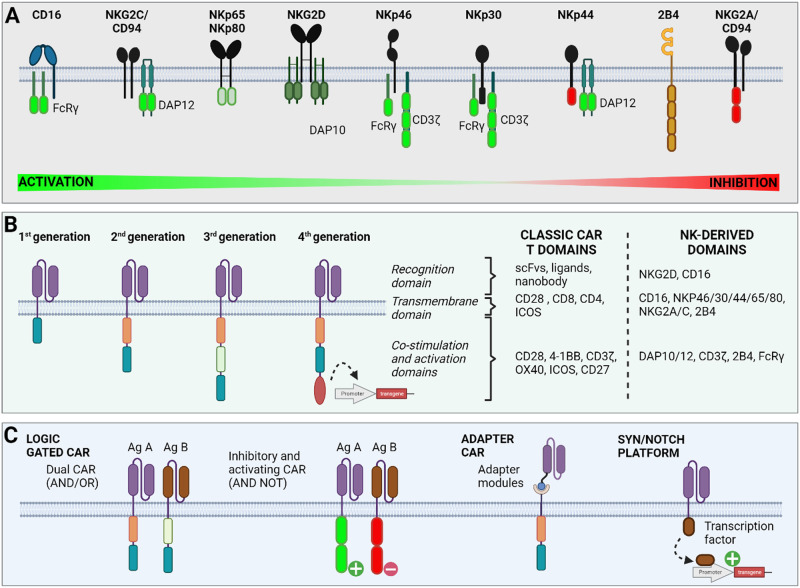
Structure of endogenous chimeric antigen receptors used in NK cell adoptive therapies. **A** NK cells harbor different activating or inhibitory receptors that directly contain activating (green) or inhibitory domains (red) or are associated with coreceptors required for signaling, such as DAP10/12 or CD3ζ. **B** Several generations of CAR constructs with recognition domains fused to transmembrane domains and intracellular signaling domains have been designed. Depending on the generation, one or multiple intracellular domains can be combined (either based on T-cell receptor domains or specifically derived from NK cell receptors to activate cells and enhance functionality). In the fourth generation, a transgene encoding cytokines is also inserted and placed under the regulation of an NFAT-sensitive promoter, which is activated upon recognition of the antigen by the CAR construct. **C** Logic-gated synthetic circuits, such as dual CARs or inhibitory CARs, have been combined in CAR-NK cells. Innovative approaches, including adapter CARs and Syn/notch receptors, are also compatible with the use of NK cells

In recent decades, adoptive cell therapies have emerged as promising approaches for harnessing the endogenous cytotoxicity of immune cells and destroying malignant cells. Among immune cell candidates, natural killer (NK) cells have drawn significant attention due to their unique ability to recognize and eliminate target cells without antigen-specific activation [1]. NK cells, which account for 5–10% of circulating peripheral blood mononuclear cells (PBMCs), are integral components of the innate immune system. These cells are classified into two distinct subsets, CD56 and CD16, based on the relative expression of surface markers. The CD56^bright^CD16^low/-^ subset is predominantly immunomodulatory and contributes to cytokine production, while CD56^dim^CD16^+^ NK cells are characterized by robust cytotoxic activity [2]. These cells exhibit cytotoxic properties similar to those of CD8 T cells but lack the CD3/T-cell receptor (TCR) complex. With the advent of single-cell RNA sequencing and multiparametric flow cytometry, novel NK cell subpopulations beyond the usual classification have been identified based on CD16 and CD56 [3, 4]. Among them, a subset called adaptive NK cells, which are characterized by a lack of Fc–epsilon receptor Ig (FCER1G) expression and an overexpression of natural killer group 2 C (NKG2C), exhibit enhanced properties, including high proliferation, increased expansion and cytotoxic potential. Unlike T cells, NK cells are not restricted to major histocompatibility complex (MHC, also called human leukocyte antigen (HLA)) recognition, and their activation requires multiple receptors. Indeed, their activation relies on finely tuning inhibitory and activating signals. To prevent the undesirable activation of healthy cells, NK cells express killer cell immunoglobulin-like receptors (KIRs) and the natural killer group 2 A (NKG2A)/CD94 heterodimer, which interacts with HLA class I molecules to provide inhibitory signals (Fig. 1). Conversely, NK cells possess an array of activating receptors, including natural killer group 2D (NKG2D), DNAX accessory molecule-1 (DNAM-1), and natural cytotoxicity receptors (NCRs), such as NKp30, NKp44, and NKp46 (Fig. 1) [2]. Upon activation by target cells, NK cells trigger multiple mechanisms to eliminate target cells, which induces the release of cytoplasmic granules loaded with perforin and granzyme B within the immunological synapse, thereby inducing the lysis of target cells. Notably, CD107a expression is upregulated following NK cell activation and is positively correlated with cytokine secretion and cytotoxic NK cell activity. NK cells can also mediate antibody-dependent cell cytotoxicity (ADCC) after tumor-bound antibody recognition by the low-affinity receptor for the constant fragment (Fc) portion of IgG1 antibodies (FcγRIIIa or CD16), thereby initiating cell death pathways [5]. Finally, NK cells can exert their cytotoxic potential through the expression of crucial death ligands, including Fas ligand (FasL) and tumor necrosis factor (TNF)-related apoptosis-inducing ligand (TRAIL), leading to target cell apoptosis after interacting with their receptor.

Considering their ability to directly kill tumor cells, adoptive transfer of NK cells has been tested in patients with advanced cancer. Here, we provide a summary of the challenges of NK cell therapies in initial clinical trials and how receptor engineering strategies have been developed to address these challenges.

## Lessons from initial clinical trials with NK cells

Several NK cell sources have been used for adoptive transfer. The NK-92 cell line has been approved for clinical application by the Food and Drug Administration (FDA) [6]. Although this cell line displays cytotoxicity against tumor cells, it has poor ADCC potential because it lacks the CD16 receptor [7]. Moreover, NK-92 cells require irradiation for safe administration, thus limiting their potency and in vivo persistence [8]. Alternatively, primary NK cells can be obtained or derived from multiple sources, including peripheral blood (PB), cord blood (CB), and induced pluripotent stem cells (iPSCs) [9]. However, ex vivo expansion is often necessary before engraftment to obtain enough NK cells, which remains a challenge. Indeed, high doses and multiple injections are often needed. Several priming techniques have been tested to not only amplify NK cells but also to enhance their cytotoxic potential [10]. However, many cytokines are added to the culture medium to render cells ‘cytokine addicted’, which impairs their in vivo persistence and expansion. Therefore, the source of NK cells, as well as the priming conditions, should be carefully chosen for adoptive transfer. Moreover, cryopreservation has also been shown to impact in vivo expansion [11].

Initially, NK cell transfer was performed in combination with hematopoietic stem cell transplantation (HSCT) for the treatment of hematological malignancies. Alternatively, NK cells were also injected without HSCT into acute leukemia patients with mitigated results. Overall, fewer than 30% of patients in different clinical trials achieved complete remission [12–14]. More encouragingly, NK cell transfer led to the eradication of measurable residual disease in two patients with acute myeloid leukemia [15]. Heterogeneous results have also been observed in non-Hodgkin lymphoma (NHL) patients, with NK cell transfer having significant clinical activity in heavily pretreated patients with advanced NHL in one report but no effect in HSCT-treated patients in another study [16, 17]. In addition to hematologic tumors, NK cell therapy has also been used to treat solid tumors, including ovarian and breast cancers, with complete remission observed in less than 25% of patients [18, 19]. In most trials, the clinical benefits often lasted only a few months before relapse, likely due to resistance or to low NK cell persistence. NK cell persistence typically ranges from a few days to 4 months, with an average of 7 days. NK cell expansion was highly variable, with no NK cell expansion observed in some trials [12, 17]. This heterogeneity may arise from the different levels of anti-tumor responses, the type of cancer studied, the stage of the disease, and interindividual differences.

A major advantage of NK cell therapy is its excellent safety profile. Compared to classical αβ T cells, NK cell transfer has several advantages, including a lower risk of inducing neurotoxicity or cytokine release syndrome (CRS) and the possibility of injecting allogeneic cells. Indeed, NK cells do not induce graft-versus-host disease (GvHD), which is mainly mediated by αβ T-cell receptor (TCR) recognition of foreign HLA-peptide complexes, and spare healthy tissue by signaling through iKIRs and NKG2A [20]. Furthermore, KIR mismatches have been shown to increase antitumoral responses. Thus, NK cells from healthy donors can be safely used for cancer therapy, even in the case of HLA mismatch between the donor and recipient, thus allowing for the generation of ‘off-the-shelf’ products. In most cases, NK cell transfers are safe and well tolerated, with low-grade and reversible treatment-related toxicity. However, high-grade adverse events have been sporadically observed, especially when NK cells were co-administered with multiple conventional therapeutic agents; this variation in results has prevented clinicians from drawing conclusions. NK cell transfer has been evaluated in combination with monoclonal antibodies. Indeed, the addition of monoclonal antibodies can potentiate NK-mediated ADCC. Allogeneic NK cells were tested in combination with the anti-CD20 monoclonal antibody rituximab in two clinical trials. The treatment was well tolerated, but only 4/15 patients and 5/9 patients in each trial achieved objective clinical responses [21, 22]. A combination of NK cell transfer and monoclonal antibodies, including anti-PD1, anti-EGFR and anti-GD2, has also shown some clinical benefits [23–28]. However, complete remission was rarely achieved, likely due to the advanced stage of the disease. Notably, two studies reported adverse events, including thrombocytopenia, myelosuppression and encephalopathy [25, 27].

Although the beneficial role of NK cells in cancer therapy lies in their ability to directly kill cancer cells, their full potential has frequently been impeded by the locally immunosuppressive and hypoxic tumor microenvironment (TME), which might explain the inconclusive clinical trial outcomes. Indeed, the TME can prevent NK cell infiltration and proliferation and can hinder activating signals. To address these limitations, engineering approaches have been proposed to enhance NK cell capacities prior to adoptive transfer [29]. Although lentiviral transduction has emerged as a prominent strategy, the commonly used vesicular stomatitis virus glycoprotein (VSV-G) pseudotype is not sufficiently effective at modifying NK cells, especially primary cells [30]. This limitation is partially due to intracellular defenses against viral components. Thus, new efficient and standardized protocols are needed. To address this problem, the incorporation of RD114 glycoproteins in lentiviral particles in conjunction with Vectofusin-1 significantly increased NK cell transduction efficacy [31]. Alternatively, mRNA/DNA electroporation offers a versatile platform to induce gene expression in NK cells. Recently, the expansion of genetic editing techniques, such as the clustered regularly interspaced short palindromic repeats/caspase 9 (Crispr/Cas9) system, has offered new perspectives for precise genetic customization.

Among these engineering strategies, membrane-anchored ectopic receptors for adoptive NK cell therapy have been developed to overcome tumor immune evasion by enhancing recognition and cytotoxicity, as well as promoting persistence, infiltration and resistance to the TME.

## Improving tumor cell recognition

### Endowing NK cells with chimeric antigen receptors

One of the most widespread immune receptor engineering approaches is chimeric antigen receptor (CAR) T cells, which involve reprogramming effector killer T cells with CARs that target membrane tumor antigens. These receptors recognize antigens through fragments of specific antibody variable chains (scFvs), which are fused to T-cell signaling and costimulatory domains. Several generations of CAR constructs with different intracellular signaling domains have been designed (Fig. 1). Initially, first-generation CARs featured only the intracellular CD3ζ stimulatory domain. Subsequent generations introduced additional costimulatory domains, such as 4-1BB and/or CD28, thereby enhancing their potential. Several CAR-T cells have been approved by the FDA for the treatment of hematological malignancies [32]. Compared with T cells, NK cells are interesting candidates for CAR therapies due to their natural ability to kill cancer cells without relying on pre-sensitization, the lower risk of cytokine release syndrome (CRS) [33, 34] and the possibility of using these cells in an allogeneic setting. Many studies have investigated the potential application of CAR-NK cell therapies both in preclinical and clinical settings. Since the first clinical trial involving CD33-CAR NK-92 cells in patients with relapsed or refractory acute myeloid leukemia [35], many clinical trials involving NK cells derived from cell lines, peripheral blood (PB) or cord blood (CB) have been launched, with a focus on hematological malignancies and certain solid tumors (Table 1). Although most trials are ongoing, some have already provided promising results. Among them, FT596, an iPSC-derived off-the-shelf CD19-directed CAR-NK cell product, induced objective responses in 5/8 patients receiving monotherapy and 4/9 patients receiving combination therapy with the anti-CD20 antibodies rituximab or obinutuzumab after the first FT506 treatment cycle. At single-dose levels of ≥90 million cells, 8 of 11 (73%) evaluable patients displayed an objective response, including 7 with complete remissions without major adverse events in a reported dose-escalation phase I study [36, 37]. Similarly, NK cells engineered with a CD19-CAR, IL15, and inducible caspase 9 suicide genes exhibited no severe treatment-related toxicity and led to an objective response in 8 out of 11 (73%) patients with B-cell lymphoid malignancies [38]. More recently, a similar engineering strategy involving an anti-CD19 CAR and IL-15 was tested in umbilical cord blood-derived NK cells and led to an objective response in 18 out of 37 (48.6%) patients with CD19 + B-cell malignancies in a phase 1/2 trial [39].

**Table 1:** Clinical trials evaluating CAR-NK cells

	CAR Target	NK source	Target cancers	Date	Phase	Status	Number
Solid tumors	5T4 Oncofetal Trophoblast Glycoprotein	Not disclosed	Advanced Solid Tumors	2022	I	Recruiting	NCT05194709
	CD70	CB-derived NK cells	Advanced Renal Cell Carcinoma, Mesothelioma and Osteosarcoma	2023	I/II	Recruiting	NCT05703854
	CLDN6	Patient-PB-NK cells	CLDN6-positive Advanced Solid Tumors	2022	I/II	Recruiting	NCT05410717
	DLL3	Not disclosed	Extensive Stage Small Cell Lung Cancer	2022	I	Recruiting	NCT05487651
	HER2	NK cell line	Recurrent HER2-positive Glioblastoma	2017	I	Unknown	NCT03383978
	Mesothelin	Autologous	Refractory Epithelial Ovarian Carcinoma	2021	0	Recruiting	ChiCTR2100048100
		iPSC-derived NK cells	Epithelial Ovarian Cancer	2018	I	Unknown	NCT03692637
	Muc1	Not disclosed	MUC1-Positive Relapsed or Refractory Solid Tumor	2016	I/II	Unknown.	NCT02839954
	NKG2D ligands	Not disclosed	Platinum-Resistant Recurrent Ovarian Cancer	2023		Recruiting	NCT05776355
		Not disclosed	Refractory Metastatic Colorectal Cancer	2022	I	Recruiting	NCT05213195
		Not disclosed	Metastatic Solid Tumors	2017	I	Completed	NCT03415100
	Not disclosed	Not disclosed	Ovarian epithelial carcinoma	2023	I	Not yet recruiting	NCT05856643
		Not disclosed	Advanced Hepatocellular Carcinoma	2023	I	Not yet recruiting	NCT05845502
		Not disclosed	Advanced Triple Negative Breast Cancer	2023	I	Not yet recruiting	NCT05686720
	PDL1	NK cell line	Recurrent/Metastatic Gastric or Head and Neck Cancer	2021	II	Recruiting	NCT04847466
	PSMA	iPSC-derived NK cells	Metastatic Castration-Resistant Prostate Cancer	2018	I	Unknown	NCT03692663
	ROBO1	Not disclosed	Pancreatic Cancer	2019	I/II	Recruiting	NCT03941457
		Not disclosed	Solid Tumors	2019	I/II	Recruiting	NCT03940820
		Not disclosed	Malignant Tumor	2019	I/II	Recruiting	NCT03931720
	TROP2	CB-derived NK cells	Platinum Resistant Ovarian Cancer, Mesonephric-like Adenocarcinoma, and Pancreatic Cancer	2023	I/II	Not yet recruiting	NCT05922930
Hematologic malignancies	CD33	Not disclosed	Relapsed/Refractory Acute Myeloid Leukemia	2021	I	Not yet ecruiting	NCT05008575
		NK cell line	Relapsed/Refractory Acute Myeloid Leukemias	2016	I/II	Unknown	NCT02944162
	CD33/TIM3	CB-derived NK cells	Acute Myeloid Leukemia	2021	0	Recruiting	ChiCTR2100043081
	CD33/CLL1	Not disclosed	Relapsed/Refractory Acute Myeloid Leukemia	2021	0	Recruiting	ChiCTR2100047084
	CD33/CCL1	Not disclosed	Acute Myeloid Leukemia	2020	I	Recruiting	NCT05215015
	CD19	Not disclosed	Relapsed/Refractory Diffuse Large B-Cell Lymphoma	2023	I	Not yet recruiting	NCT05673447
		iPSCs-derived NK cells	CD19-positive B-Cell Malignancies	2023	I	Recruiting	NCT05336409
		Allogenic	B-Cell Hematologic Malignancies	2022	I/II	Recruiting	NCT05654038
		Allogenic	Adult Relapsed/Refractory B-cell Malignancies	2022	I	Recruiting	NCT05645601
		Not disclosed	Relapsed/Refractory Acute Lymphoblastic Leukemia	2022	I	Recruitment completed	NCT05563545
		CB-derived NK cells	Refractory/Relapsed B-cell Non-Hodgkin Lymphoma L	2022	I	Recruiting	NCT05472558
		Not disclosed	Relapsed/Refractory B-cell Malignancies	2022	I	Recruiting	NCT05410041
		Allogenic	B-cell Malignancies	2021	I	Recruiting	NCT05020678
		HLA haploidentical NK cells (PB)	Refractory/Relapsed B-cell Non-Hodgkin Lymphoma	2021	I	Recruiting	NCT04887012
		CB-derived NK cells	B Lymphoid Malignancies	2021	I	Recruiting	NCT04796675
		Not disclosed	Relapsed/refractory B-cell Acute Lymphoblastic Leukemia	2021	I	Recruiting	NCT05379647
		iPSC-derived NK cells	B-cell Lymphoma or Chronic Lymphocytic Leukemia.	2020	I	Recruiting	NCT04245722
		Not disclosed	Relapsed or Refractory B-Cell Non-Hodgkin Lymphoma	2020	I	Not yet ecruiting	NCT04639739
		Not disclosed	Relapsed and Refractory B-Cell Lymphoma	2019	I	Unknown	NCT03824951
		iPSC-derived NK cells	Relapsed and Refractory B-Cell Lymphoma	2018	I	Unknown	NCT03690310
		UC and CB-derived NK cells	B Lymphoid Malignancies	2017	I/II	Completed [38]	NCT03056339
		NK cell line	CD19 Positive Leukemia and Lymphoma	2016	I/II	Unknown	NCT02892695
		PB NK cells (allogenic)	Relapsed and Refractory Lymphoma	2013	I	Completed.	NCT01974479
		PB NK cells (allogenic)	Relapsed and/or Refractory Acute Lymphoid Leukemias	2009	I	Completed.	NCT00995137
	CD19/CD22	iPSC-derived NK cells	Relapsed and Refractory B-Cell Lymphoma	2019	I	Not yet recruiting.	NCT03824964
	CD19/CD70	CB-derived NK cells	Refractory/Relapsed B-cell Non-Hodgkin Lymphoma	2023	I/II	Recruiting	NCT05842707
		CB-derived NK cells	Refractory/Relapsed B-cell Non-Hodgkin Lymphoma	2022	I	Recruiting	NCT05667155
	CD70	CB-derived NK cells	Relapse/Refractory Hematological Malignances	2021	I/II	Recruiting	NCT05092451
	CD7	Allogenic	CD7 Hematologic Malignancies	2022	I	Not yet recruiting	NCT05377827
		NK cell line	CD7 Positive Relapsed or Refractory Leukemia and Lymphoma	2016	I/II	Unknown	NCT02742727
		Not disclosed	Hematological Malignancies	2023	I	Recruiting	NCT05995028
	CD123	Allogenic	Refractory/Relapsed Acute Myeloid Leukemia	2022	I	Recruiting	NCT05574608
		Not disclosed	Refractory/Relapsed Acute Myeloid Leukemia and Blastic Plasmacytoid Dendritic Cell Neoplasm	2023	I/II	Recruiting	NCT06006403
	CD5 ( + IL15)	CB-derived NK cells	Relapse/Refractory Hematological Malignances	2021	I/II	Recruiting	NCT05110742
	CD56	Not disclosed	Relapsed/Refractory NK/T-cell lymphoma/NK cell leukemia	2023	II	Recruiting	NCT05941156
	CD20	iPSC-derived NK cells	Relapsed/Refractory Acute Myelogenous Leukemia and B-Cell Lymphoma	2019	I	Active	NCT04023071
	CD22	iPSC-derived NK cells	Relapsed and Refractory B-Cell Lymphoma	2018	I	Unknown	NCT03692767
	CD33/CLL1	Not disclosed	Acute Myeloid Leukemia	2023	I	Not yet recruiting	NCT05987696
	CCL1	iPSC-derived NK cells	Acute Myeloid Leukemia	2023	I	Recruiting	NCT06027853
	CD38/SLAMF7	iPSC-derived NK cells	Acute myeloid leukemia and multiple myeloma	2020	I	Active	NCT04614636
	BCMA	Allogenic	Relapsed/Refractory Multiple Myeloma or Plasma Cell Leukemia	2023	I	Recruiting	NCT06045091
		Allogenic	Relapsed/Refractory Multiple Myeloma	2022	I	Recruiting	NCT05652530
		iPSCs-derived NK cells	Multiple myeloma	2021	I	Recruiting	NCT05182073
		UC and CB-derived NK cells	Relapse/Refractory Multiple Myeloma	2021	I	Recruiting	NCT05008536
		NK cell line	Relapse/Refractory Multiple Myeloma	2019	I/II	Recruiting	NCT03940833
		iPSC-derived NK cells	Relapsed/Refractory B-Cell Lymphoma	2018	I	Unknown	NCT03559764
	NKG2D ligands	Not disclosed	Relapsed/Refractory Acute Myeloid Leukemia	2023		Recruiting	NCT05734898
		CB-derived NK cells	Relapsed/Refractory Acute Myeloid Leukemia	2022		Recruitment completed	NCT05247957
		PB NK cells	Myelodysplastic Syndromes *and Acute Myeloid Leukemia*	2020	I	Recruiting	NCT04623944
	Not disclosed	Not disclosed	B-Cell Malignancies	2021	I/II	Recruiting	NCT04747093
		Not disclosed	Relapsed/Refractory Hematological Malignancies	2021	I	Recruiting	NCT04796688
Other	NKG2D ligands – SARS-CoV-2 envelope glycoprotein	CB-derived NK cells	COVID-19	2020	I/II	Recruiting	NCT04324996

#### Classical CAR constructs

First-generation CARs composed of the CD3ζ chain [40, 41] and second-generation CARs composed of CD28 and CD3ζ [38, 42–44], 4-1BB and CD28 [45], 4-1BB and CD3ζ [46, 47], 4-1BB, CD28 and CD3ζ [48], or CD137 and CD3ζ [49, 50] were applied to redirect NK cells against tumor cells. Several antigens were targeted by the CARs, including CD19, CD20, CD33 and CD5. Alternatively, the efficacy of second- and third-generation CAR-NK cells has been explored for several solid tumors, including ovarian and breast cancers, as well as glioblastoma, with promising results [51–56]. Indeed, cytotoxic responses against tumor cells expressing the target antigen were triggered following coculture with CAR-NK cells [51–54]. This CAR-NK cell-mediated killing was specific, as no or only a minimal effect on healthy tissue was observed [51, 54]. Furthermore, this efficacy was confirmed in preclinical studies in which CAR-NK cells controlled tumor growth and improved survival rates [51–54]. For instance, in xenograft models of glioblastoma and ovarian cancers, the survival of tumor-bearing mice was significantly prolonged after CAR-NK cell treatment, which was associated with a decrease in tumor volume [52, 54]. Similar results were obtained in a humanized mouse model of nasopharyngeal carcinoma [53].

#### CAR constructs with NK receptor domains

In addition to domains derived from receptors expressed in T cells, domains from NK cell receptors have also been incorporated into CAR constructs to improve NK cell activation (Fig. 1). Several NK cell receptors have been shown to replace the extracellular recognition domain, the transmembrane region and/or the intracellular region of CAR receptors. Overall, many CAR configurations were assessed in NK cells with various combinations of transmembrane regions, including CD16, NKp44, NKp46, and NKG2D, as well as costimulatory domains, such as 2B4, DAP10, DAP12, or 4-1BB, either alone or combined with CD3ζ. However, the benefits of incorporating NK cell-associated domains over conventional domains in CARs have not been systematically investigated.

Notably, the extracellular CAR was replaced by the NKG2D receptor, thus allowing for NK cell activation in response to the detection of NKG2D ligands [57–59]. The transmembrane NKG2D receptor has also been exploited in several CAR constructs to promote its association with co-signaling receptors and mediate NK cell activation [60–62]. Alternatively, NK cells expressing CARs with both transmembrane and intracellular CD16 regions were shown to effectively target lung adenocarcinoma tumors in vivo [63]. Incorporating the intracellular activating domains of NK cells has also proven effective. For instance, some CARs have been designed with intracellular regions based on the activating receptors 2B4 [44, 60, 62, 64–66] and NKG2D [61], the adapter proteins DAP10 [57] and DAP12 [59, 67, 68], or the Fc receptors FcεRγ [69] and CD16 [63].

Some researchers have attempted to compare the effects of NK-derived receptor domains with those of T-cell-derived receptor domains in CAR constructs. For instance, the 2B4 region (alone or with DNAM1) promoted the expansion and persistence of CAR-NK cells, in contrast to CD28 administration or the absence of a costimulatory signal [65]. The addition of 2B4 domains instead of 4-1BB also resulted in greater cytotoxicity [70]. This observation was confirmed in a paper comparing the cytotoxicity of a CAR construct composed of the CD28 homolog, 2B4 and CD3ζ domains, versus that of a CAR with CD28, 4-1BB and CD3ζ intracellular domains [71]. The cytotoxic effect of CAR-NK cells with the DAP12 intracellular domain was also enhanced compared to that with the CD3ζ signaling chain [67]. In addition to their effects on intracellular domains, modifications of the transmembrane domain have also been reported to impact CAR-NK cell functionality. For instance, the use of the NKG2D transmembrane domain combined with 4-1BB enhanced the responses of NK cells compared to a CAR harboring the CD8 transmembrane domain and the 2B4 activation region [72]. The effect of replacing the commonly used scFv with an NK receptor recognition domain was also investigated. For instance, the use of an scFv targeting the NKG2D ligands MICa/b led to an increased antitumoral response compared to the insertion of the NKG2D extracellular domain [73].

Overall, NK receptor-based domains are valuable for improving CAR designs, and optimizing domain combinations may be beneficial in the future. To enhance tumor cell recognition and immune responses by engineered NK cells, NK cell activating receptors can also be used in combination with CAR receptors. Notably, bispecific CAR-NK cells engineered with a PD1-DAP10 CAR and NKG2D exhibited strong cytotoxicity against gastric cancer cells both in vitro and in vivo [74]. Indeed, anti-PD1-DAP10 CAR harnessed NKG2D signaling through the DAP10 domains and was shown to potentiate NK cell activation against PD1 and NKG2D ligand-expressing tumor cells [74].

#### Next-generation CAR constructs

More sophisticated approaches, including the fourth CAR-T-cell generation, have emerged in recent years (Fig. 1). An IL-15-encoding transgene was inserted under the control of an NFAT responsive element, along with a CAR construct targeting CD44 [75]. Upon recognition of the target antigen by the CAR, a signaling cascade was initiated, leading to NFAT activation and the secretion of IL-15, which improved NK cell cytotoxicity in a triple-negative breast tumor spheroid model [75]. In addition, the incorporation of proinflammatory or proliferation-inducing cytokine transgenes may also enhance the T-cell response.

Further improvements were based on engineering CARs targeting multiple antigens to counteract tumor escape (Fig. 1). Indeed, cancers are highly heterogeneous and resist targeted therapies via antigen escape. Many examples of target antigen loss after CAR-T-cell therapy, including melanocyte differentiation antigen [76], CD19 [77], CD22 [77] and BCMA [78], have been described. To allow for the concomitant detection of several tumor markers, NK cells were transfected with mRNAs encoding both CD19 and BCMA CARs. The cytotoxic effects of NK cells expressing both CARs outperformed those of single CAR-expressing NK cells [79]. Similarly, dual CAR constructs that integrate two distinct antigen recognition domains have been developed in T cells and might be tested soon in NK cells. This approach may increase the stringency of tumor recognition and prevent tumor evasion (i.e., even if one antigen is lost, the other antigen will still be detected by the second CAR, and it is less likely that both antigens will be lost simultaneously) [80]. Notably, as NK cells can detect tumor cells through many activating endogenous receptors, even if the CAR target antigen is lost, cytotoxic responses may still occur through the recognition of other ligands, contrary to CAR-T cells. Adapter CARs, which are derived from traditional CARs, are also particularly useful for detecting multiple antigens because they provide greater flexibility in antigen targeting (Fig. 1). The unique feature of an adapter CAR is the inclusion of an adapter module between the antigen recognition domain and the signaling domain. This adapter module acts as a bridge to connect the antigen binding process to the subsequent activation of immune signaling pathways. Such adapter CARs have been implemented in NK cells, such as an anti-FITC CAR combined with a FITC-folate adapter that links CAR and folate receptor alpha-expressing breast tumor cells [72]. Similarly, a universal CAR recognizing 2,4-dinitrophenyl (DNP) was redirected in NK cells to target several epitopes of gp160 using DNP-conjugated antibodies as adapter molecules [81].

More complex logic-gated CARs have been developed in which the activation of therapeutic effects is controlled by logical combinations of input signals, thus allowing more specific and adaptable targeting of cancer cells (Fig. 1). This is particularly important for improving the discrimination between diseased and healthy tissues. An ‘OR and NOT’ logic-gated CAR gene circuit approach was designed to target CD33- or FLT3-expressing acute myeloid leukemia tumor cells while sparing healthy hematopoietic stem cells. This strategy was achieved through the insertion of two activating CARs targeting FLT3 and CD33 (or a single bivalent CAR targeting both antigens) and an EMCN-specific inhibitory CAR in NK cells [82]. NK cells were protected from fratricide by using an NK self-recognizing inhibitory CAR along with an activating CAR [83]. Notably, even if NK cells endogenously express the activating CAR ligand, this target antigen can also be transferred, following binding with CAR receptors, from target tumor cells to effector NK cells through a process called trogocytosis [84]. Trogocytosis can dampen CAR-NK cell activity due to a decrease in tumor antigen density at the tumor cell surface but can also lead to fratricides after self-recognition and continuous CAR engagement [83, 85]. In this way, the inhibitory CAR provides a ‘don’t eat me’ signal, thereby preventing NK cell fratricide. In the future, complex logic-gated circuits may enable the dynamic and precise activation of immune responses against cancer cells, thus offering a more controlled and personalized approach to cancer immunotherapy. In addition, the Syn/notch receptor was used in NK92 cells to trigger the expression of IL-12 following glypican-3 (GP3) recognition [86]. Similarly, a logic-gated GPC3 Syn/notch receptor was used to control the expression of a CAR targeting CD147 in a hepatocellular carcinoma model, leading to the specific killing of GPC3 + CD147+ cells but not single antigen-positive cells [9, 87]. The Syn/notch receptor is composed of an extracellular recognition domain, the transmembrane domain of the Notch core protein and an intracellular transcription factor. Upon binding the target antigen, the transcription factor is released and can activate the expression of downstream genes. This platform is entirely programmable in terms of recognition and effector transgenes, thus offering multiple possibilities for NK cell reprogramming. This programmability will be crucial for generating modular responses, which can evolve with the disease and counteract tumor escape. Even if autonomous circuit regulation is the ultimate goal, complex logic gates exogenously controlled by user-provided cues have been developed for T cells. The SUPRA CAR platform consists of a split CAR system to switch on/off the target antigen without the need for an additional CAR-T-cell injection [88]. This switch should effectively counteract antigenic loss through the targeting of multiple antigens. Moreover, SUPRA CARs also offer tunable signaling strength, which can decrease the risk of overactivation and thus of serious adverse events. Even if SUPRA CARs have not yet been tested in NK cells, they are theoretically compatible. Similarly, VIPER CARs (versatile protease regulatable CARs) can be controlled using FDA-approved antiviral protease inhibitors, which can induce an ON or OFF CAR state [89]. Interestingly, VIPER CARs can be multiplexed to generate complex circuits, which can be transposed to NK cells. FDA-approved small molecule inducers can also be used to control therapeutic responses at the transcription level. For instance, synthetic zinc finger transcription regulators (synZIFTRs) are composed of a DNA-binding domain, a drug-sensitive domain, and a transcription factor [90]. Upon drug exposure, user-controlled activation of target genes has been achieved in T cells. Furthermore, this approach might be suitable for activating several cellular programs that drive synergistic responses. Overall, such platforms should help refine the NK cell responses of each patient at a given time point depending on the stage of disease. In the future, artificial intelligence (AI)-guided tools might be extremely useful for adapting these therapies based on defined disease biomarkers.

#### Combinations of CARs with other therapies

Treating patients by CAR-NK cell transfer alone may not be sufficient because of the advanced stage of the disease and because NK cells are inhibited in the targeted environment (such as the TME). Combining CAR-NK cell transfer with conventional therapies such as chemotherapy and radiotherapy could potentiate efficacy. For instance, radiotherapy is useful not only for directly destroying tumor cells but also for reshaping the TME, notably by modifying the vasculature and cytokine secretion, which promote engineered immune cell infiltration, expansion and activation. The synergistic effect of CARs and monoclonal antibodies that target either activating receptors, such as CD16 [91], immune checkpoint inhibitors [92], such as PDL1 and PD1, or tumor antigens [93], was also observed. These antibodies can be externally produced by direct injection or produced directly by genetically engineering NK cells [91] or tumor cells [94]. In addition to antibody transgenes, cytokine transgenes were also co-introduced with CARs in NK cells to improve persistence (as described below). In addition, the cytotoxicity of CAR-NK cells increased following their combination with oncolytic viruses in a brain metastasis mouse model [95]. These combination treatments underline the value of synergistic CAR-based approaches to trigger more efficient responses against various malignancies by enhancing not only NK cell activation but also NK cell recruitment and infiltration in the TME.

### Endowing NK cells with ectopic TCRs

Although most strategies have focused on CAR-NK cells, the threshold of antigens required for NK cell activation is relatively high (Table 2). Moreover, the use of such receptors does not allow the targeting of intracellular antigens, except if the scFv recognizes an HLA-peptide complex. Indeed, this was described with a TCR-like CAR that was specific for a mutated nucleophosmin-1-derived neoepitope presented by HLA-A2 in an acute myeloid leukemia model [96]. Intracellular antigens are processed before being presented on the MHC cell surface. These peptides are then recognized by specific TCRs expressed by T cells. The classical TCR complex is composed of TCRα and β chains and four CD3 signaling chains (one ε, one γ and two ζ). TCR engineering has mainly been applied to T cells [97], but the expression of the endogenous TCR (unless it is knocked out) can lead to chain mispairing and chimeras between endogenous and ectopic TCR chains, thus impairing TCR recognition and function. As NK cells do not harbor an endogenous TCR, there is no risk of mispairing after TCR insertion. However, the expression of a functional TCR in NK cells is more complex than that in T cells. The introduction of transgenes encoding the α and β TCR chains is not sufficient because the sequences encoding the CD3 signaling chains are also required for TCR stabilization at the membrane and for downstream signaling [98]. Notably, only the insertion of CD3ζ is unnecessary since NK cells naturally express this subunit intracellularly. Other major components are missing in most NK subsets, such as the CD8β chain, which is known to impact the functionality and signaling of many TCRs and is often inserted along with the TCR and CD3 chains in NK cells. Recently, the feasibility of TCR-reprogramming strategies was demonstrated in the FDA-approved NK cell line NK-92, leading to degranulation, cytokine production, and tumor cell killing both in vitro and in vivo [98, 99]. Similar results were observed in another NK cell line, the YTS cell line, with a TCR that targeted an antigen associated with melanoma [100]. Importantly, the addition of a new TCR did not impair the endogenous functions of NKs. For instance, engineered NK cells can still be inhibited by self-components and mediate activation through other receptors (CD16 and NKG2D) [100]. Although NK cell lines are the main source of NK cells for the development of TCR NKs, in one study, researchers successfully reprogrammed human primary NK cells. A BOB1-specific TCR along with the CD3 subunits was introduced by retroviral transduction in primary NK cells, leading to antigen-specific cytotoxicity and cytokine production by NK-TCR cells to a level comparable to that of CD8 T cells expressing the same TCR [101]. Moreover, after injection in a multiple myeloma mouse model, a reduction in tumor growth was observed [101]. This finding offers interesting perspectives for clinical testing in upcoming years. However, NK-TCR therapies may be hampered by the poor presentation of tumor-associated antigens, which may not reach a sufficient level to mediate NK cell activation. Interestingly, TCR affinity and avidity are similar in primary T cells and in NK cell lines, suggesting that the characteristics of a given TCR could be transposed and used to optimize responses in NK cells [102]. CAR and TCR can also be combined to obtain a synergistic effect. For instance, NK cells have been engineered with a TCR specific to the E7 protein of HPV16 jointly with a CAR specific to TROP2 [103]. The CAR construct comprised two costimulatory domains (CD28 and 4-1BB) but lacked the CD3ζ activating domain; thus, CAR activation was necessary but not sufficient for NK cell-mediated cytotoxicity. Indeed, NK cell activation is dependent on E7 epitope recognition, which is exclusively expressed in HPV16-infected tumor cells, and cytotoxicity is enhanced by the binding of CAR to the TROP2 target expressed mostly on tumor cells but also in some healthy tissues. Combined signaling leads to optimal and tumor-specific cytotoxicity while sparing healthy tissues [103]. This strategy lays the foundation for sophisticated logic gates and broader applications in the future. For instance, instead of using two distinct receptors (one CAR and one TCR), hybrid receptors may be constructed. Such receptors, either HLA-independent (HIT receptor [104] and STAR [105]) or HLA-dependent (TCAR [106]), have already been developed in T cells and may be transposed to NK cells. Notably, a chimeric TCR composed of the extracellular domains of the TCRα chain fused to the CD28 transmembrane domain followed by the 2B4 and DAP10 signaling domains and the TCRβ chain fused to the CD28 transmembrane domain followed by the 41BB and CD3*ζ* signaling domains has been constructed [107]. After expression in primary NK cells, antigen-specific recognition and the lysis of tumor cells were achieved both in vitro and in vivo [107].

**Table 2:** Comparison of CAR- and TCR-engineered NK cells

	CAR	TCR
HLA restricted	No	Yes
Antigen density required	High	Low
Intracellular targeting	No	Yes
Multiplexing	Yes	Yes
Off the shelf	Yes	Yes
Clinical test	Yes	No

Overall, TCR-engineered NK cells exhibit unique advantages, by allowing the recognition of intracellular antigens, with lower levels required for activation and no risk of mispairing.

## Enhancing the ADCC potential of NK cells

In addition to the direct recognition of tumor cells by dedicated receptors, NK cells can mediate target cell destruction through the coordinated action of antibodies via ADCC. Indeed, antigen-specific antibodies bind and opsonize target cells, and their constant region is then detected by Fc receptors expressed on NK cells. Following recognition, NK cells trigger target cell lysis through the release of lytic enzymes. FcγRIII (also known as CD16) has two allelic variants, with either a phenylalanine (F) or a valine (V) at amino acid position 158. These variants give rise to Fc receptor isoforms with different affinities: low affinity (CD16a-158-F/F) and high affinity (CD16a-158-V/V). Due to its high affinity, the CD16a-158-V/V variant has mainly been used for NK engineering to promote ADCC [108, 109]. For instance, by combining high-affinity CD16 V158 FcγRIIIa receptor and IL-2 expression in engineered NK92 cells, enhanced lysis of tumor cells was observed with diverse combinations of monoclonal antibodies and target cells [7]. The therapeutic efficacy of high-affinity CD16a overexpression in CD38 KO NK cells was confirmed in vivo in a multiple myeloma model after adoptive transfer and treatment with a daratumumab antibody [110]. Importantly, to potentiate efficacy, FcR-engineered NK cells are often combined with monoclonal antibodies targeting a tumor-associated antigen. A similar NK reprogramming approach was successfully developed to mediate the ADCC of PDL1-expressing meningioma tumors in the presence of an anti-PDL1 antibody [111]. One limitation of these approaches is the predisposition of CD16 to ADAM17 proteinase-mediated cleavage, which impairs ADCC. Notably, a noncleavable variant (i.e., resistant to proteinase) has been developed and successfully implemented, which had improved killing in preclinical models [112–114].

High-affinity CD16 receptors have also been expressed with CAR receptors in NKs. Notably, granule polarization and the release of lytic enzymes were observed in NK92 cells expressing the high-affinity variant and a CAR [115]. Alternatively, as mentioned above in Section 3.1, the CD16 domain can be directly incorporated into a CAR construct as a transmembrane and/or costimulatory domain [63]. Conversely, to potentiate ADCC, the extracellular recognition domain of CD16a can be fused to signaling and activation domains. Indeed, the common CD28 and 4-1BB domains used in CARs have been linked to CD16a, either alone or with the signaling domain of CD3ζ [45]. The resulting chimeric receptor exhibits enhanced cytotoxic activity and ADCC activity against CD20-positive tumor cells in the presence of a monoclonal antibody [45]. NK-specific signaling domains (2B4, FcRγ and DAP10) have also been fused with the CD16a recognition domain [116]. More complex chimeric receptors based on FcR domains have also been constructed. For instance, the ASIMut (antigen-specific synthetic immunoglobulin-based multichain) receptor is composed of two partners: a membrane-anchored immunoglobulin fused to FcεRIγ and the extracellular and transmembrane regions of Igα and Igβ linked to the CD3ε signaling domain [117]. After co-culturing modified NK cells and target cell lysis, ADCC-like lysis of target cells was induced [117].

Overall, the targeted engineering of NK cell receptors holds great potential for enhancing ADCC and thereby improving the precision and efficacy of immune-mediated NK cancer therapies.

## Improving NK cell chemotropism

A significant limitation of current adoptive cell therapies is poor infiltration into tumors, which limits effectiveness [118]. Although certain chemokine receptors are expressed on freshly isolated NK cells, this expression is attenuated following ex vivo expansion or freezing. Some ex vivo culture conditions have emerged as promising strategies to significantly upregulate the expression of key receptors, such as C-X-C motif chemokine (CXCR) 3, which is known to enhance the migratory capacity of CXCL10-producing tumor and immune cells [119]. This unregulated expression allows for the selection, amplification, and upregulated expression of receptors before NK cell infusion. However, even optimized culture conditions often do not induce the expression of sufficient levels of chemokine receptors and are not efficient for all chemokine receptors. Thus, the genetic engineering of NK cells to integrate ectopic chemokine receptors that enhance their chemotropism may be more relevant for enhancing the efficacy of adoptive therapies for cancers (Table 3).

**Table 3:** Chemokine receptor engineering to improve NK cell migration

Receptor	Target	NK sources	Modification techniques	Target disease	Outcomes	Ref
CXCR1	IL-8	PB	mRNA electroporation	Ovarian cancer	Greater chemiotaxis in vivo Increased tumor control	[123]
CXCR2	CXCL5	PB	Retroviral vector	Renal cell carcinoma	Greater chemiotaxis in vitro Increase target cell killing and adhesion in vitro	[120]
CXCR2	CXCL1-3 and CXCL5-8	NK92	CRISPR-Cas9	Human Colon Cancer	Greater chemiotaxis in vivo into tumor sites Stronger cell-killing and proliferation activity Tumor reduction Increased survival	[122]
CCR2B and CCR4	CCL22 or CCL2	NK-92 and PB	Lentiviral vector	None	Greater chemiotaxis in vitro	[130]
CXCR4	CXCL12 and SDF-1α	YTS	Lentiviral vector	Glioblastoma	Greater chemiotaxis in vitro and in vivo Tumor reduction/clearance Increased survival	[68]
CXCR4	SDF-1α	PB	Lentiviral vector	None	Greater chemiotaxis in vitro	[126]
CXCR4^R334X^	SDF-1α	PB	mRNA transfection	None	Greater chemotaxis in vitro Increased the bone marrow homing	[125]
CXCR4 and CCR7	CXCL12 and CCL21	NK92	Lentiviral vector	Colorectal cancers	Tumor reduction Increased survival	[127]
CCR5	CCL5	PB	Lentiviral vector	Human Colon Cancer	Greater chemiotaxis in vitro and in vivo	[132]
CCR7	CCL19 and CCL21	PB	Trogocytosis	None	Greater chemotaxis in vitro Increased the lymph node homing	[129]
CCR7	CCL19 and CCL21	NK-92	DNA transfection	B-cell lymphoma	Greater chemiotaxis in vitro and in vivo Increased tumor control Increased survival	[128]
CCR7	CCL19	PB	mRNA electroporation	None	Greater chemiotaxis in vitro	[109]

Several receptors have been shown to improve NK cell migration toward tumor sites. For instance, CXCR2, which is lost upon in vitro expansion, was overexpressed in NK cells by retroviral transduction, leading to enhanced migration toward renal cell carcinoma spheroids [120]. Similar results were observed in vivo, as the overexpression of CXCR2 on NK-92 cells promoted their infiltration into lung metastases [121]. Alternatively, the CRISPR/Cas9 system has been used to upregulate the expression of chemokine receptors such as CXCR2 and IL-2 on NK cells, thereby leading to increased migration to tumor sites, enhanced proliferation and improved cell killing in a preclinical colon cancer model [122]. The modulation of CXCR1 has also demonstrated encouraging results. Modified NK cells engineered with CXCR1 and a CAR targeting tumor-associated NKG2D ligands were reported to enhance migration toward tumors and increase infiltration in vivo [123]. Using CRISPR gene editing, CCR5 was also effectively overexpressed, while CXCR4 expression was downregulated in expanded NK cells, resulting in reduced trafficking in the liver and increased levels in circulation [124]. In contrast, for hematologic malignancies affecting the bone marrow, upregulation of CXCR4 expression is beneficial for improving homing to tumor cells. Indeed, the retention of NK cells in the bone marrow is mediated by CXCR4 through interactions with its ligands SIP5 (sphingosine-1 phosphate receptor 5) and CXCL12, also known as SDF-1 (stromal cell–derived factor 1). For instance, mRNA transfection with a gain-of-function variant of CXCR4 (CXCR4^R334X^) significantly promoted the homing of NK cells to the bone marrow following adoptive transfer [125]. CXCR4 overexpression has also been combined with CARs targeting either CD19 or epidermal growth factor (EGFR)vIII in NK cells to enhance migration toward tumor cells and the retention of endogenous and CAR-mediated cytotoxic activities [68, 126]. Combined overexpression of CXCR4 and C-C motif chemokine receptor (CCR) 7 via lentiviral vectors further enhanced NK cell migration to human colon cells [127]. The insertion of CCR7 alone into NK cells also promoted their migration toward secondary lymphoid organs. Through mRNA electroporation, CCR7 was effectively introduced into primary human NK cells, leading to a substantial improvement in their in vitro migration toward CCL19 [109]. Similarly, transfection of a nonviral vector containing the CCR7 receptor, along with a high-affinity CD16 Fc receptor and an anti-CD19 CAR, resulted in enhanced survival and superior tumor control in humanized mice bearing lymphoma tumors [128]. Trogocytosis-mediated expression of CCR7 on expanded NK cells also led to NK cell migration toward CCL19 and CCL21 both in vitro and in vivo (toward lymph nodes) [129]. Notably, other receptors not expressed on NK cells, such as CCR4 and CCR2B, which are naturally found on regulatory T cells and tumor-resident monocytes, were used to reprogram NK cells [130].

Some strategies have also been developed to promote chemokine secretion, such as IFNγ injection, in combination with oncolytic viruses or radiotherapy, to facilitate NK cell recruitment [119, 131]. Alternatively, chemokines can be artificially expressed in the TME. For instance, combination approaches, such as modifying NK cells to express CCR5 while introducing a vaccinia virus expressing the CCL5 chemokine, have been successfully applied to enhance migration [132].

Although most approaches have focused on chemokine receptors, other receptors, such as the E-prostanoid 3 receptor, led to increased cell adhesion and cytotoxicity in modified NK cells and should be further tested [133].

## Improving NK cell persistence and proliferation

The proliferation and persistence of NK cells following adoptive transfer display distinct dynamics depending on their cellular source. While irradiation of NK cell lines before transfer impacts their in vivo persistence, primary NK cells retain a degree of physiological relevance and familiarity with the host environment, thereby enhancing their persistence after transfer. However, this persistence remains limited to a couple of weeks, thus requiring frequent injections. To increase NK cell survival and growth ex vivo, cytokine cocktails, including those with IL-2, IL-12, IL-15, IL-18, and IL-27, have been developed. Through the meticulous modulation of growth factors, cytokines, and signaling molecules, NK cells can be endowed with memory-like properties and have greater responsiveness upon re-exposure to antigens, prolonged persistence, and augmented cytotoxicity.

Among these factors, IL-15 was shown to promote NK cell proliferation and activation while exerting a low level of toxicity [134, 135]. The administration of this cytokine has been extensively exploited as a means of increasing the longevity of NK cells. In addition to direct IL-15 injections, NK cells harboring an IL-15 transgene have been engineered to survive in the absence of exogenous cytokines in an autocrine manner [136]. The addition of the IL-15 gene notably prolonged CAR-NK cell persistence and cytotoxicity [38, 44, 137, 138]. In this context, very promising results were obtained in a phase I/II clinical trial evaluating HLA-mismatched anti-CD19 CAR-NK cells transduced with a retroviral vector encoding IL-15 in lymphoid tumors. Among the first 11 reported patients, 8 (73%) achieved a response, including 7 who achieved complete remission. Expansion was observed as early as 3 days after infusion, and CAR-NK cells persisted at low levels for at least 12 months [38]. Given that IL-15 is secreted, fusion proteins were constructed to anchor IL-15 at the membrane to ensure direct action on NK cells [139]. The integration of a membrane-bound IL-15 with a chimeric NKG2D receptor fused with costimulatory (OX40) and signaling (CD3ζ) domains in NK cells amplified cytotoxicity, prolonged intrinsic longevity, and enhanced antitumor efficacy in acute myeloid leukemia xenograft models, thus highlighting its value as a potent therapeutic agent [139]. In addition, a membrane-bound IL-15/IL-15R fusion protein was extensively tested in combination with CAR in NK cells [140]. For instance, BCMA CARs, along with noncleavable CD16 receptors and monoclonal anti-CD138 antibodies, were combined with this fusion molecule in NK cells and promoted in vitro cytotoxicity and prolonged persistence in the modified cells [114, 141]. Interestingly, this fusion approach was evaluated in a clinical trial with an anti-CD19 CAR and a noncleavable CD16 receptor (along with monoclonal antibodies) and led to partial responses in diffuse large B-cell lymphoma patients [37]. Alternatively, other fusion proteins have been developed, notably a chimeric protein composed of IL15 and the CD8α transmembrane domain [142]. In leukemia and sarcoma mouse models, NK cells engineered to express this membrane-bound IL-15 exhibited elevated cytotoxicity both in vitro and in vivo [142].

Another strategy to promote survival in the absence of exogenous cytokines is to combine signaling domains that induce proliferation with unrelated recognition domains. For instance, truncated IL-2 receptor β-chain (ΔIL-2RB) and STAT3-binding tyrosine-X-X-glutamine (YXXQ) motifs were added to anti-PD-L1 CARs to enhance cell proliferation and persistence through the activation of downstream signaling pathways [72].

In conclusion, the modulation of cytokines and cytokine receptors is highly promising for enhancing NK cell survival and persistence, thus paving the way for durable therapeutic efficacy.

## Counteracting the immunosuppressive tumor microenvironment

Achieving successful outcomes with NK cell therapies hinges not only on the capacity of immune cells to migrate and persist within the intricate TME but also on their ability to remain functional despite the immunosuppressive milieu.

**Fig. 2:**
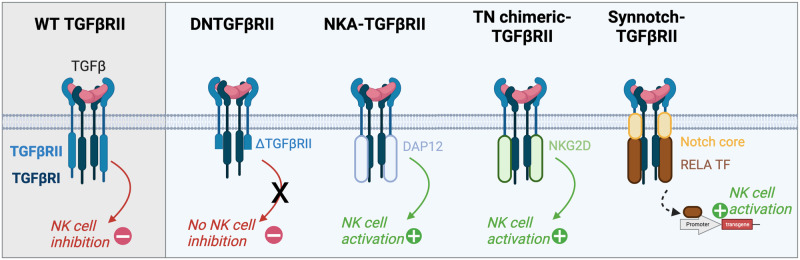
Hijacking the TGFβRII receptor promotes NK cell activation. Upon the recognition of TGFβ by the wild-type TGFβRII receptor, a signaling cascade is initiated, resulting in a compromised NK cell phenotype and reduced cytotoxicity. A dominant-negative mutant in which most of the intracellular domain of TGFβRII was deleted abrogated signaling in response to TGFβ, thus preventing NK cell inhibition. This intracellular region was also replaced by the intracellular domains of DAP12 and NKG2D, which mediate NK cell activation upon TGFβ recognition by the extracellular regions of TGFβRII. The Syn/notch platform was combined with TGFβRII: the transmembrane region of this receptor was fused to the Notch core domain and to the RELA transcription factor on the intracellular region. Upon recognition of TGFβ, a mechanical force is triggered, leading to the release of the RELA transcription factor, which induces genes involved in NK cell activation

Transforming growth factor-beta (TGF-β) plays a pivotal role in the TME by suppressing NK cell-mediated immune responses and promoting tumor immune escape. TGF-β inhibits the expression of activating receptors, such as NKG2D and DNAM1, thereby impairing NK cell recognition and the targeting of tumor cells. TGF-β is recognized by a complex formed by the TGFβRI and TGFβRII receptors and induces intracellular pathways leading to NK cell inhibition (Fig. 2). To suppress this inhibition, the intracellular part of the TGFβRII receptor was deleted to generate a dominant-negative mutant (DNTβRII). After expression in NK cells, DNTβRII protected NK cells from TGF-β-mediated immunosuppression, while they retained their killing ability after NK stimulation (Fig. 2) [143–145]. Indeed, following stimulation with the endogenous NKG2D or DNMA1 receptor or with an ectopic CAR receptor, cytolytic activity, and notably the secretion of perforin, granzyme and IFN-γ [144, 145], was preserved. These results were confirmed in vivo. The transfer of genetically modified NK-92 cells that incorporated DNTβRII decreased tumor proliferation and lung metastasis and increased the survival rate in a mouse model [146]. Technological advancements have been made to encompass the synthetic Notch-like receptor (Syn/Notch), as in “NKCT,” where the truncated TGFβRII domain is fused to the Notch transmembrane domain coupled to the RELA transcription factor (Fig. 2). This fusion protein promoted the direct activation of NK cells at the transcription level, thereby boosting their resistance to the TME and augmenting their antitumor functions [147]. Other chimeric receptors that integrate activating domains and a TGFβRII recognition domain have exhibited promising results in overcoming TGF-β immunosuppression. Among them, engineered NK-92 cells expressing a hybrid receptor (named TN-chimeric), which is composed of the extracellular part of TGFβRII and the transmembrane and intracellular domains of NKG2D, exhibited enhanced chemoattraction to tumor cells expressing TGF-β and had enhanced killing ability (Fig. 2) [148]. A similar construct in which the intracellular domain was replaced by a DAP12 NK activation motif successfully initiated NK cell activation following exposure to TGF-β (Fig. 2) [147]. Notably, other activating domains, such as the activating domains of proinflammatory cytokine receptors, which have already been validated in T cells, may be suitable for redirecting TGF-β activity [149, 150].

In addition to anti-inflammatory cytokines, immune checkpoint inhibitors, such as PD-1/PD-L1, also play a major role in immune cell inhibition by the TME. Strategies similar to those used for TGF-β, such as the development of truncated receptors, the replacement of the receptor intracellular domain with an activating unit or the use of anti-PD-L1 CARs (as mentioned above in Section 3.1), have been shown to reverse PD1/PDL1-mediated inhibition [53, 61, 69, 151]. Indeed, the use of truncated PD-1 in transferred NK92 cells led to reduced tumor growth and improved survival rates [152]. The recently developed NK cell-specific PD1-based chimeric switch receptor (PD1-CSR) uses the DAP10, DAP12, and CD3ζ signaling domains to reverse the effect of PD1 on NK cells [74, 153]. After administration in patients, an increase in NK cell degranulation and cytokine expression against autologous CD138^+^PD-L1^+^ malignant plasma cells was observed [153]. Collectively, these innovative strategies highlight the growing complexity of adoptive cell therapy in reprogramming NK cells to overcome the challenges of the endogenous TME and promote antitumor immune responses.

## Decreasing the expression of endogenous NK receptors

NK cells can be inhibited by the tumor microenvironment and have also been reported to kill each other. To avoid fratricide, knockouts (KOs) of target genes have been generated. For instance, in the treatment of hematologic malignancies, the CD38 and CD7 genes were deleted in engineered CAR-NK cells [40, 114, 154, 155]. Interestingly, a 2-in-1 strategy was developed by inserting the CAR receptor in the locus of the gene encoding its target, which allowed for the expression of the CAR while avoiding fratricide [156, 157]. As mentioned above in Section 3.1, inhibitory CAR receptors can also be implemented to avoid trogocytosis-mediated CAR-NK cell fratricides [83, 85].

In addition, in the case of allogenic transfer, donor HLA-mismatched NK cells can be eliminated by the recipient immune system. To counteract this “host-versus-graft effect”, the surface expression of HLA class I molecules on donor NK cells was abolished by beta 2-microglobulin KO to prevent killing by recipient alloreactive CD8^+^ T cells. Because loss of HLA class I molecules induces killing by NK cells (‘missing self’-induced lysis), a single-chain HLA-E molecule was also introduced to inhibit receiver NK cells expressing CD94/NKG2A or CD94/NKG2B [158].

Given that NK cell activation relies on a balance between signals from activating and inhibitory receptors, disrupting signals from inhibitory receptors is also expected to promote cell activation without increasing the activity of activating receptors. Strategies to counteract the impact of inhibitory signaling pathways, including KO or downregulation of the expression of these inhibitory receptors, have been explored [50, 159].

## Conclusions and perspectives

NK cell receptor engineering holds great promise for reprogramming immune responses to cancer cells. Nevertheless, some major limitations remain to be addressed, such as the persistence of infused cells, which is quite limited and often does not allow for the establishment of memory responses. By carefully choosing the donor and the phenotype of infused NK cells, such as cytokine-induced memory-like NK cells or adaptive NK cells, prolonged and durable effects can be achieved [160–163]. In addition, with persistence in patients, engineered NK cells should also be functional and exert therapeutic benefits. In addition to inhibition by the TME, other mechanisms of resistance, such as upregulated HLA-G expression on tumor cells, have been observed following CAR-NK cell therapy and should be taken into consideration [66]. A strategy to boost the efficacy of NK cell therapy is to combine engineered targets. In this context, AI will be useful for identifying receptors, coreceptors, or checkpoint inhibitor KO combinations to generate next-generation NK cell therapies. For instance, cytokine-inducible SH2-containing (CISH) disruption was shown to potentiate the antitumor activity of CAR-NK cells [164, 165]. In addition to multiple engineering methods, AI can help develop combination treatments with conventional treatments or other immunotherapies by predicting patient responses. Notably, cell engagers have gained much interest in recent years and have been approved for clinical use. They can bridge ligands, such as tumor antigens, and activate receptors on the NK cell surface [166–172]. This kind of molecule could be used to promote NK cell activation by linking engineered receptors to tumor cells. Importantly, safety issues remain intrinsically related to engineered NK cell therapies, such as off-target activation. Ensuring the specificity of NK cell therapies is essential to avoid damaging healthy tissue, which can lead to serious side effects. To refine NK cell activation and selectively target cancer cells, tightly tuned logic-gated circuits should be developed. Moreover, computational models should be developed to predict and dynamically adjust cell responses. The frequent use of integrative vectors to modify NK cells also comes with the risk of insertional mutagenesis, which may lead to NK cell lymphomas. The current FDA recommendation is to have a vector copy number less than 5 [173]. Moreover, editing tools can also be used to generate chromosomal translocations. Recently, several manufacturing protocols have been developed to overcome some of the abovementioned limitations and, if approved, could become standard practices to minimize chromosome loss and translocations in manufactured products [174–176]. As a safeguard, to ensure engineered cell destruction in the event of adverse events, an inducible caspase safety switch should be implemented [38, 177]. Quality control standards must be defined to check the engineering efficacy, purity, phenotype and tumorigenicity of NK cells. Indeed, depending on the donor, the source (PB or UCB) and the culture conditions, NK cell subsets can vary [178]. For these latter points, new techniques to expand, modify and cryopreserve primary NK cells ex vivo would be extremely useful for scaling up production for clinical application, as high doses and multiple injections are required [33]. The future progress achieved in T-cell therapies, including the infrastructures developed for the production of cell and viral vectors, as well as the tools available to engineer cells (among which in vivo editing strategies), are expected to synergistically improve NK cell therapy. Early-stage clinical trials will also provide valuable information and promote optimization for future NK cell therapies, which are likely to reach clinical approval in the coming years. Overall, these pioneering advancements not only hold tremendous potential for cancer treatment but also extend the spectrum of treatments for other diseases, including infections.
